# Integration of a Blog into an Emergency Medicine Residency Curriculum

**DOI:** 10.5811/westjem.2015.8.27199

**Published:** 2015-11-12

**Authors:** Jay Khadpe, James Willis, Mark A. Silverberg, Andrew Grock, Teresa Smith

**Affiliations:** SUNY Downstate Medical Center, Department of Emergency Medicine, Brooklyn, New York

## BACKGROUND

Technologies and techniques for knowledge translation are rapidly evolving and there is a need for graduate medical education (GME) curricula to keep up with these advances to reach our learners in an effective manner. Technologies such as blogs, microblogs, wikis, podcasts, and vodcasts have the potential to expand upon the current didactic models by adding dimensions and engaging learners in modalities not previously available.^1^

Emergency medicine (EM) has been at the forefront in adopting social media in the pursuit of knowledge and collaboration.^2^,^3^ In order to advise residencies on how to use these new technologies, the Council of EM Residency Directors (CORD) Social Media Task Force published its guidelines, best practices, and recommendations for integrating social media into EM residency programs laying the foundation for EM residency-based social media activities.^4^ In March 2012, “The Original Kings of County” (TOKC) blog was launched in an effort to integrate social media into the SUNY Downstate/Kings County Hospital EM Residency Program (Figure). The blog represents an early adapter of these efforts and uniquely applies them at the GME level to improve learner engagement with the EM residency curriculum.

## EDUCATIONAL OBJECTIVES

1. To increase resident engagement in their didactic curriculum through use of a residency blog.
2. To develop residents’ skills with respect to education and scholarship through authorship for a residency blog.

## CURRICULAR DESIGN

The TOKC blog was implemented to create an online hub for the integration of social media into the residency curriculum at the SUNY Downstate / Kings County Hospital EM residency. Three overarching goals drive the content for the blog. The first is to post educational content that mirrors didactics already occurring within the program to reinforce the material and share it with residents who are unable to attend these activities. Examples include our Morning Reports that are typically brief case discussions with clinical pearls written by senior residents and a “Wednesday Wrap-up” that summarizes learning points and resources related to topics discussed during weekly conference didactics. By posting these resources on the blog, the impact of these didactics is magnified and enables all learners to benefit.

The second goal is to engage residents through their own authorship of “featured” blog posts. This activity often coincides with the residents’ development of an academic niche as they hone their skills as academic authors. We have used a mixed editorial process with respect to these posts decided between the author and blog editors. In some cases, a faculty editor aids with post development prior to publication; and in other cases, the resident will post directly to the blog and post-publication review will take place through the comment section. By allowing for both editorial formats, residents were more enthusiastic about authoring and contributing to the blog.

The third goal is intended to attract and encourage participation in the blog. Clinical cases are posted for residents to interpret and discuss. The resident who submits the most accurate and inclusive interpretation, as judged by the author and faculty editor, receives a small prize, such as a gift card. For example, residents rotating in the coronary care unit are asked to submit an electrocardiogram, which is then reviewed by a resident author and faculty editor prior to posting. These competitions are an attempt to embrace the competitive spirit among residents together with a monetary award to drive learners to the blog who might otherwise not participate; and once at the site, engage them with the other available content. Overall, this three-prong approach creates a comprehensive online didactic presence that embraces the use of social media to promote learning.

## IMPACT/EFFECTIVENESS

Since launching, TOKC has generated over 600 posts by more than 20 resident and faculty authors as of April 2015. It currently receives more than 100 page views per day. This provides our program a platform to share their scholarship with a local, national, and international community. Alumni authors of TOKC have gone on to blog for internationally recognized academic EM blogs, such as *Academic Life in EM*, and to lecture at national conferences on how to use social media for medical education, demonstrating how participation in a residency-based blog can aid in the development of an academic niche. Additionally, TOKC was highlighted in the article, “Integration of Social Media in EM Residency Curriculum,” by Scott et al. published in *Annals of EM* as a model for integrating a blog into an EM residency program.^5^

## Figures and Tables

**Figure:**
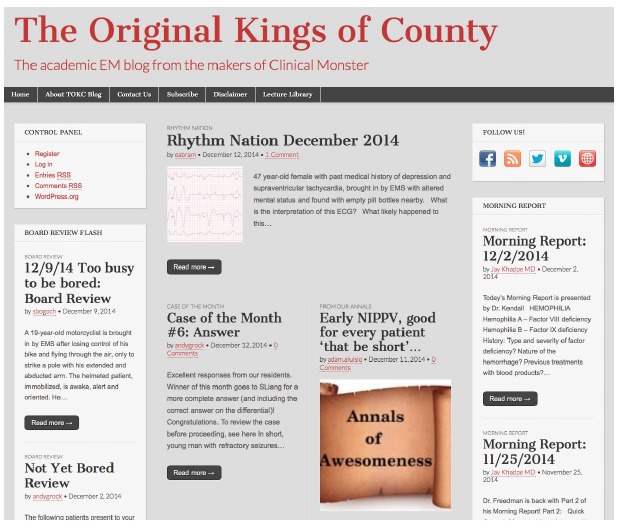
The front page of The Original Kings of County blog.
